# Single-cell sequencing revealed the microglia-activated cell distribution in gray matter heterotopia

**DOI:** 10.1016/j.gendis.2024.101235

**Published:** 2024-02-01

**Authors:** Penghu Wei, Chunhao Shen, Jinkun Xu, Quanlei Liu, Yihe Wang, Xiaotong Fan, Yongzhi Shan, Guoguang Zhao

**Affiliations:** a Department of Neurosurgery, Capital Medical University, Xuanwu Hospital, Beijing 100053, China; b Clinical Research Center for Epilepsy, Capital Medical University, Beijing 100053, China; c Beijing Municipal Geriatric Medical Research Center, Beijing 100053, China

Gray matter heterotopia (GMH) is caused by malformation of cortical development, which exhibits as gray matter nodules abnormally located within the white matter region, and most GMH patients have concomitant epilepsy. Although antiepileptic drugs can control seizures, over 40% of patients still require surgery to pursue a cure for the disease. Previous researchers have studied GMH from the genetic perspective, and several disease-specific gene modifications were identified to elucidate the pathogenesis.^1^ However, to acquire a deeper understanding of the disease progression, it is essential to comprehensively explore the transcriptional alterations caused by GMH. Here, we sought to analyze the cell types and their distinct transcriptional changes in GMH. Thus, we studied the lesional tissues from GMH patients using single-cell RNA sequencing, which captured the cell-type specific transcriptome at single-cell resolution.

We collected the GMH tissues and the controls from two patients undergoing surgical resection for epilepsy (Table S1). A schematic of the lesional area was presented using 3D reconstruction (Fig. 1A). GMH samples with controls were sent for single-cell RNA sequencing workflow, and 18,661 cells were captured. Following quality-control filtering, we collected 16,475 cells (Fig. 1B). The heatmap visually displayed the genes that were specifically expressed in different cell types (Fig. 1C). Uniform manifold approximation and projection (UMAP) was employed to identify 11 major cell lineages based on their markers: microglia (*ADORA3*, *HLA-DRA*, and *SIGLEC8*), oligodendrocytes (*CNP*), neutrophils (*FCGR3B*), endothelial cells, oligodendrocyte progenitor cells (*TNR*), fibroblasts (*PDGFRB*), astrocytes (*GFAP*), natural killer cells (*NKG7*), monocytes (*CD163*), epithelial cells (*CD34*), and smooth muscle cells (*ACTA2*). Gene feature plots then validated the precision of cell annotation^2^ (Fig. 1D, E). We also used UMAP to display the distinct cell types found in GMH alongside their controls (Fig. S2A). Microglia was the cell type with the highest proportion (60.2%), followed by oligodendrocytes (15.8%), neutrophils (10.7%), endothelial cells (3.6%), oligodendrocyte progenitor cells (3.3%), fibroblasts (2.1%), astrocytes (1.3%), *etc* (Fig. S2B). The percentage of microglia increased from 57.46% to 62.91%, while that of oligodendrocytes increased from 13.26% to 18.21% in GMH (Fig. S2C).

**Figure 1:**
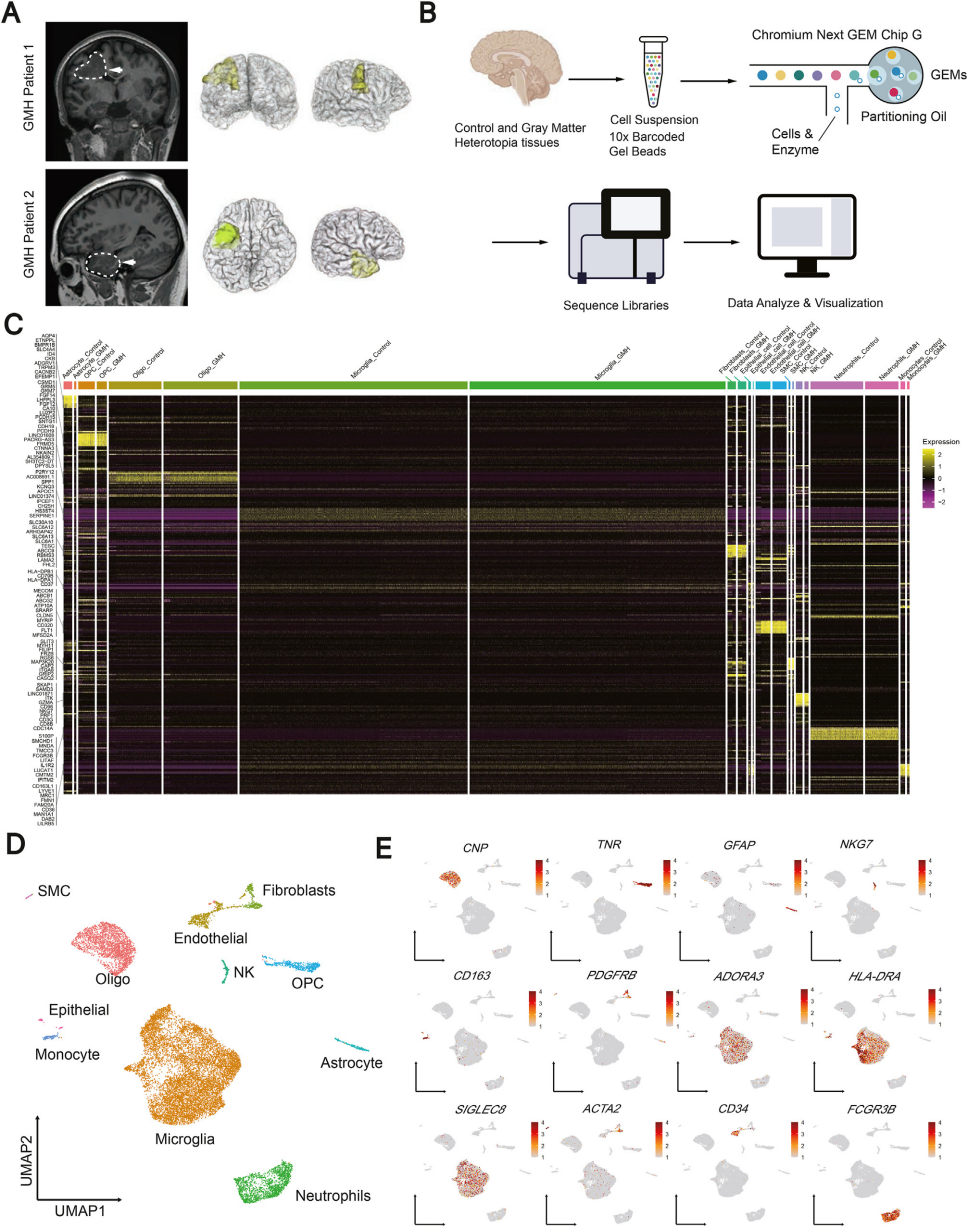
Single-cell RNA sequencing of excised tissues from patients with gray matter heterotopia (GMH). **(A)** The 3D scan reconstruction of the GMH area showing the exact location of the resected tissue in the human brain. **(B)** The schematic diagram showing the single-cell RNA sequencing workflow. **(C)** The heatmap showing the cell-type-specific gene expression in GMH and the control. **(D)** The UMAP map showing the whole cells captured from GMH samples and the control. **(E)** The UMAP plots showing the typical expression of marker genes for identifying different cell populations. UMAP, uniform manifold approximation and projection.

We further analyzed the differential expressed genes (DEGs) (*P* < 0.05, |Log_FC| > 0.5) for each cell type (Fig. S2D). The DEGs in microglia exhibited the most significant transcriptional alteration (Fig. S2D). Gene ontology enrichment analysis revealed that the top 10 terms in microglia are related to the regulation of immune cells' migration, activation, and downstream signaling pathways (Fig. S2E). GMH microglia exhibited a significant expression of several DEGs, including *CD46*, *RIPK2*, *SYK*, and *HLA-DRB5*, which are associated with immunological activation. In contrast, *TLR4* and *PDPN* in the control group exhibited normal cell migration and adhesion (Fig. S2E, F). To confirm the inflammatory properties of microglia, we conducted TSA multicolor immunofluorescence staining. Microglia were categorized into two phenotypes, which induced or combatted the inflammation based on their related gene expression.^3^ We observed the significantly higher CD68 expression (pro-inflammatory marker) and the decreased CD163 expression (anti-inflammatory marker) in GMH. CXCL10 functioned in recruiting immune cells, and its levels were shown to be elevated in GMH (Fig. S2G).

Next, we identified five distinct inflammatory subgroups of microglia: the *P2RY12*^+^, *JUN*^+^, *CCL4*^+^, *APOE*^+^, and *APOC*^+^ subgroups (Fig. S3A). *P2RY12* was highly expressed at the initial stage in the microglia-associated immune process, contributing to maintaining brain homeostasis. The proportion of the *P2RY12*^+^ subgroup reduced from 16.14% to 10.86%, indicating that the inhibition of anti-inflammatory protection occurred in GMH (Fig. S3B). The increased DEGs in the *P2RY12*^+^ subgroup (*P2RY12*, *KCNQ3*, and *HS3ST4*) demonstrated a state maintaining the anti-inflammatory balance (Fig. S3C, D). An increase (40.19%–47.79%) was seen in the *JUN*^+^ subgroup. The increased expression of JUN could promote the pro-inflammatory processes and perhaps sustain the epileptic state of the brain (Fig. S3B). *APOE*^+^ and *APOC*^+^ subgroups are associated with inhibitory microglial inflammation and late-onset epilepsy.^4^ The *APOE*^+^ group in our data exhibited a minimal increase in GMH (6.33%–7.15%). Nevertheless, the *APOC*^+^ subgroup in GMH experienced a relative decrease (4.78%–2.42%) compared with the control. Overall, *APOE*^+^ and *APOC*^+^ microglia still exhibited a gene expression of microglial subgroups that promoted inflammation (Fig. S3B–D). In addition, GSEA enrichment analysis also validated the pro-inflammatory enhancement of hemostasis and signal transduction gene sets (Fig. S3E). Subsequently, we performed gene ontology enrichment analysis using DEGs of each microglia subgroup. Glial cell migration was observed in the *P2RY12*^*+*^ cluster, whereas T-cell activation and positive regulation of cytokine production were observed in the *CCL4*^*+*^ cluster. Other clusters exhibited functions related to cell adhesion (Fig. S4A–E).

To further demonstrate the process of microglial activation, a time-series trajectory study of microglia was conducted (Fig. S5A–C). The distribution of microglial subtypes along the trajectory revealed that the *P2RY12*^*+*^ cluster initially played the main role, while the *APOC*^+^ cluster was up-regulated later (Fig. S5D). This can also be confirmed by examining the differentiation trajectory tree of each subgroup (Fig. S5E). Subsequently, we investigated the manifestation of particular gene sets and the transcriptional features of microglia revealed a promotion of both microglial activation and migration (Fig. S5F).

We next sought to determine if the pro-inflammatory gene expression in microglia could explain the epileptic symptoms observed in GMH. We investigated the intercellular communication and discovered that multiple types of glial cells released epilepsy-related cytokines (Fig. S6A). The findings also revealed that cytokines such as WNT, TNF, and IGF were the specific output factors associated with GMH. These factors are known to be involved in the mTOR and MAPK signaling pathways, which are closely related to neurological inflammation.^5^ Also, microglia decreased the IL-1 expression in tissues while simultaneously showing an overexpression of specific pro-inflammatory markers. The study demonstrated that IL-6 and IL-16 exhibited an enhanced incoming signal of inflammation, while TNF demonstrated an outgoing inflammatory pathway (Fig. S6B, C). In addition, the analysis of cell interaction revealed the expression of IL1, SPP1, and PTN from the control, which was another evidence of pro-inflammation. Conversely, the presence of IL10 from GMH suggested an anti-inflammation as a response to the microglia activation. The expression of WNT in GMH was meaningful as it was confirmed to function in neuronal differentiation and the development of epilepsy (Fig. S6D).

In conclusion, we used single-cell sequencing to reveal the transcriptional alterations in GMH and enriched the DEGs to a pro-inflammatory state. We next focused on microglia and its subgroups. By doing a more in-depth analysis of five distinct microglial subtypes and their time-series transcriptional trajectories, we observed an increase in the expression of specific pro-inflammatory features. Moreover, the stimulation of pro-inflammatory microglia has a direct relationship with epilepsy-related cytokines, suggesting that heightened inflammation in brain tissue contributes to a greater likelihood of developing epileptic foci. Hence, therapies aimed at addressing the management of epilepsy should prioritize the avoidance of neuroinflammation. Pharmacological or genetic methods that can reduce localized neuroinflammatory responses may be practical approaches for controlling epilepsy.

## Ethics declaration

Patients and controls were acquired with informed consent under the protocol approved by the Ethics Committee of Xuanwu Hospital Capital Medical University (Code: KS2021118-1). No blinding was done.

## Author contributions

Guoguang Zhao and Yongzhi Shan conceived and designed the entire research; Penghu Wei, Chunhao Shen, and Jinkun Xu performed the experiments, collected the experimental data, analyzed the data, and wrote the manuscript; Quanlei Liu, Yihe Wang, and Xiaotong Fan performed bioinformatics analysis. Penghu Wei, Chunhao Shen, and Jinkun Xu contributed equally to this work. All authors approved the final version of the manuscript.

## Conflict of interests

The authors declared no potential competing interests.

## Funding

The research was supported by the National Natural Science Foundation of China (No. 82030037, 81871009, 81801288), the National Science and Technology Innovation 2030 Major Projects of China (STI2030-Major Projects, No. 2021ZD0201801), and the Translational and Application Project of Brain-inspired and Network Neuroscience on Brain Disorders, Beijing Municipal Health Commission (China) (No. 11000022T000000444685).

## Data availability

The data of this study are available on request from the corresponding authors.
