# Supplementary material for: Single-cell sequencing revealed the microglia-activated cell distribution in gray matter heterotopia
Source: Genes Dis. 2024 Feb 1;12(1):101235. doi: 10.1016/j.gendis.2024.101235 (PMC11466553; doi:10.1016/j.gendis.2024.101235)
Supplement: Multimedia component 1 [file mmc1.docx]

## Methods

### Sample Information

Details of the human brain samples are displayed in Table S1. Samples used in the experiments were obtained from patients treated with epilepsy surgery at Xuanwu Hospital Capital Medical University. Due to the irregular location of the focal area, the surgically resected tissues inevitably carried some relatively normal tissues. After surgery, the excised tissues were sent for pathological identification to separate the focal tissue from the relatively normal tissue, which we called para-focal tissue. Then, these were preserved separately for single-cell RNA sequencing. Individual consent was obtained from patients. The procedure was guided under a protocol approved by the Xuanwu Hospital Capital Medical University Ethics Committee.

### Tissue dissociation and cell suspension preparation

We used the Tissue Preservation Solution (Preservation Solution Ratio) to preserve the excised tissue and a GentleMACS™ Octo Dissociator product to dissociate the tissue. Mix Enzyme 1 was configured in the ratio of 50 µl Enzyme P, 1900 µL Buffer Z, and Mix Enzyme 2 was configured in the ratio of 20 µL Buffer Y and 10 µL Enzyme A. The neural tissue was extracted from the human brain and washed in cold D-PBS. An appropriate volume of enzyme mix 1 was prepared and transferred to a gentleMACS C tube. Using a scalpel, larger tissue (e.g., whole brain or spinal cord) was cut into approximately 8 sagittal sections or 0.5 cm pieces. This step was skipped for smaller tissue, such as the hippocampus. The tissue slices were transferred to a C-tube containing 1950 µL of Mixing Enzyme 1, and 30 µL of Mixing Enzyme 2 was transferred to the C-tube. The C-tube was closed tightly and hung upside down on the sleeve of the gentleMACS Octo Dissociator, and the appropriate gentleMACS program (20-100 mg, select 37C_ABDK_02; >100 mg, select 37C_ABDK_01) was run. After termination of the program, the C-tube was removed from the gentleMACS Octo Dissociator and centrifuged briefly to collect the sample from the bottom of the tube. The sample was resuspended and applied to a MACS SmartStrainer (70 μm) placed on a 50 mL tube. Ten milliliters of cold D-PBS were added to the C-tube, and the C-tube was closed and shaken gently. The D-PBS was added to the MACS SmartStrainer (70 μm), and the cell suspension was centrifuged for 10 min. The supernatant was aspirated, and debris removal and erythrocyte removal were performed.

### Human brain tissue cell 10x sequencing

Cell capture and cDNA synthesis were performed using a single cell 3’ library, gel bead kit V3 (10x Genomics, 1000075), and Chromium single cell B-chip kit (10x Genomics, 1000074). After loading the cell suspension (300-600 live cells per microliter as determined by Count Star) into the Chromium Single Cell Controller (10x Genomics) according to the manufacturer’s protocol, single-cell gel beads were generated in the emulsion. Finally, single cells were suspended in PBS containing 0.04% BSA. The captured cells were lysed, and the released RNA was barcoded in individual GEMs by reverse transcription.

Reverse transcription was performed on an S1000TM Touch Thermocycler (Bio-Rad) with a first-round at 53°C for 45 minutes. Then, the temperature was 85°C for 5 minutes and held at 4°C to generate cDNA. cDNA was then amplified, and quality was assessed using an Agilent 4200.

According to the manufacturer’s instructions, single-cell RNA-seq libraries were constructed using the Single Cell 3’ Library. Libraries were finalized using an Illumina Novaseq6000 sequencer with a sequencing depth of at least 100,000 reads per cell, using a paired-end 150 bp (PE150) cellranger pipeline using Cell Ranger software obtained from the 10x Genomics website (https://support.10xgenomics.com/single-cell-geneexpression/software/downloads/latest). After alignment, filtering, barcode counting, and UMI counting with the cellranger count module, the raw sequencing data were used to generate the feature-barcode matrix, determine the clusters, and output the cellular gene expression matrix.

### Multisample data integration analysis

We sequenced a total of 18661 single cells (9031 from patient 1 and 7714 from patient 2) with an average of 1946 genes captured per cell and performed quality control filters with unique characteristics of individual cells between 200~4000 and a percentage of mitochondrial genes in individual cells (percentage.mt) less than 10%.

Using sctransform, a normalization method in which cell sequencing depth is used as a covariate in a generalized linear model based on Pearson residuals from a “positive-negative binomial regression”, biological heterogeneity is preserved while eliminating the effect of technical features in downstream analysis. A weighted nearest neighbor analysis based on anchor correlation was built by identifying cell pairwise correspondences between individual cells across the dataset. The multisample data were merged by identifying shared subpopulations across the dataset through sample correspondence. The completed integrated data were subjected to linear downscaling and uniform manifold approximation and projection (UMAP) at 4000 nfeatures per sample, PCA 1:50.

### Cell type identification

After completing cluster grouping, cells were automatically annotated using the SingleR package regarding the hpca.se dataset. The cell types were manually annotated according to the cellmarker database website (https://biocc.hrbmu.edu.cn/CellMarker/) and the human brain cell marker genes in the panglaoDB database website (https://panglaodb.se). The cell types were also verified by high expression of differential genes between clusters.

### Differential gene and GO enrichment analysis

To examine the functional differences between focal and parafocal tissues, we grouped the merged datasets by cell type using FindAllmarker in the seurat package and performed differential gene screening between cells of focal and parafocal origin in each group with a fold change of 0.25 and *p* < 0.05. The output list of differential genes was analyzed for GO enrichment using the enrichGO function in the clusterProfiler package. The number of differential genes, log P value, associated genes, and pathways of the enrichment results were displayed by bar graphs, dot plots, and pathway plots.

### Cellular trajectory analysis

To investigate the process of cell differentiation and the relationship between microglial subtypes, we performed a proposed time-series analysis of microglia using the Monocle3 package to construct trajectories simulating cell changes over time by using the asynchronous progression of individual cells in an unsupervised framework. We also observed the change of some genes over ‘time’ according to the trajectory and the cell subtype classification reference.

### Analysis of intercellular communication

To investigate alterations in intercellular associative communication between focal and parafocal tissues, we analyzed cellular communication using the CellChat package. By analyzing known structures of ligand-receptor interactions in cells, such as multimeric ligand-receptor complexes, soluble agonists, antagonists, stimulatory and inhibitory membrane-bound coreceptors, cell-state-specific signaling communication in given single-cell RNA sequencing data was inferred using mass action models, as well as differential expression analysis and statistical tests on groups of cells. The analysis was used to study the altered cellular communication networks in GMH.

### TSA multicolor immunofluorescence staining

CD163 (ab182422, Abcam Technology, Cambridge, UK), CD68 (ab955, Abcam), CXCL10 (ab8098, Abcam), and IBA1 (ab48004, Abcam) were labeled for detection using the AlphaTSA 7-color fluorescent staining kit (Alphaxbio, Beijing, China) in combination with digital pathology imaging analysis technology. Paraffin sections were first placed in a 60°C thermostat and baked, dewaxed, hydrated, and then microwave-repaired with the antigen repair solution included in the kit. After that, incubation with primary and secondary antibodies, fluorescent staining, and DAPI staining were performed, and finally, image acquisition was performed after sealing the sections.

## Supplementary table

**Table S1 Clinical information of GMH patients**

|  | Patient 1 | Patient 2 |
| --- | --- | --- |
| **Gender** | Male | Female |
| **Age** | 11 years old | 29 years old |
| **Age at onset** | 10 years old | 29 years old |
| **Location** | Right frontal lobe | Right temporal lobe |
| **Symptom** | Epilepsy | Epilepsy |
| **Epileptic seizure form** | Myoclonic seizure | Focal seizures |
| **Pathological diagnosis** | Gray matter heterotopia | Gray matter heterotopia |
| **Frequency** | Occasionally | 3-4 per month |
| **Operation** | Right frontal GMH resection | Right temporal GMH resection |
| **Tissues IHC** | NeuN(+), GFAP(+), Olig-2(+),  Syn(+), Reelin(+), MAP-2(+),  NF(+), CD34, SMI-32p(+),  Ki-67(1%+) | NeuN(+), Nestin(+), Syn(+),  Reelin(+), Calretinin(+) |

## Supplementary figure legends


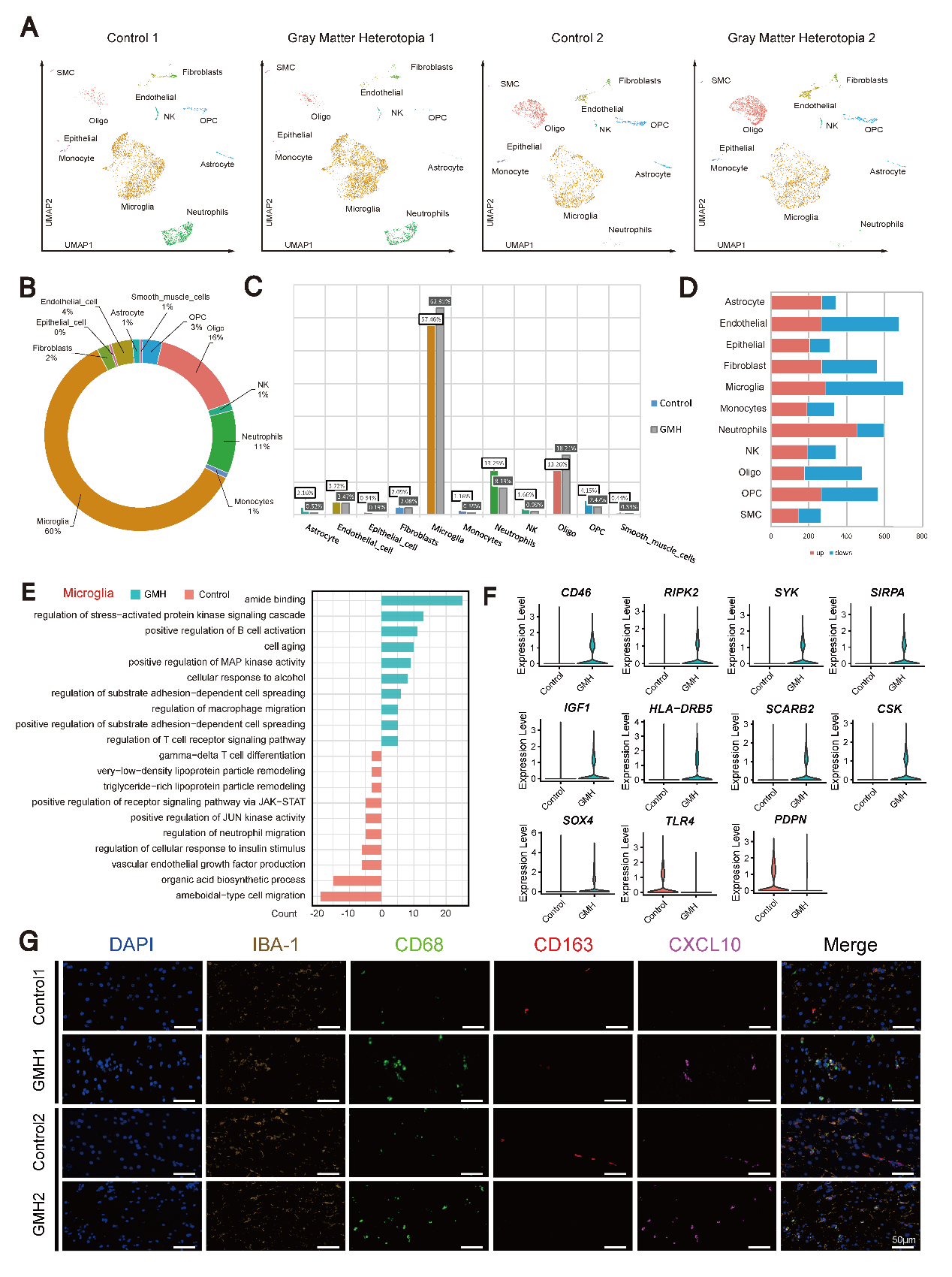


**Figure S2 Microglia contributed mainly to the cell inflammatory homeostasis changes in gray matter heterotopia.**

(**A**) UMAP visualization of cells separately in each sample. (**B**) Donut chart showing the percentage of different cell types in the merged cell population. (**C**) Bar plot revealing the percentage changes of each cels in GMH versus the control tissue. (**D**) Bar graph showing the variation in upregulated and downregulated genes in all cell types. (**E**) GO analysis unveiling the top enriched functions of microglia between GMH and control tissue. (**F**) Violin plots showing the differentially expressed genes in related GO terms. (**G**) Multicolor immunofluorescence staining showing the expression of microglia-related markers in all our samples, scale bar = 50 μm.


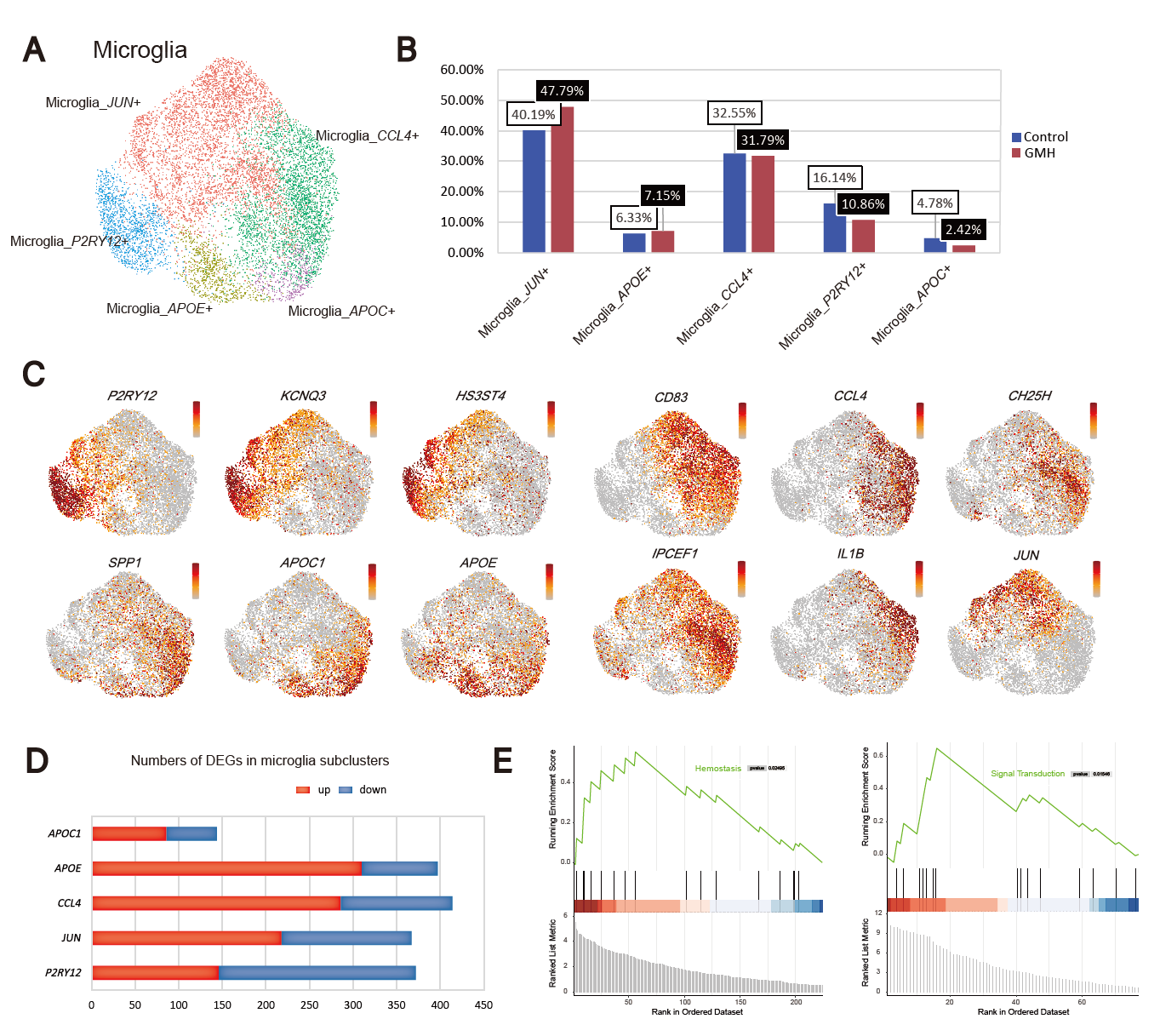


**Figure S3 Further classification of microglial clusters showing the distribution and functional enrichment.**

(**A**) UMAP plot of microglia mapping the classification of its endogenous subtype. (**B**) Bar graph showing the percentage of each microglial subtype between the GMH and control groups. (**C**) Feature plots displaying some of the marker genes with regional expression features. (**D**) Bar graph showing the calculation of upregulated and downregulated genes in different microglia subgroups. (**E**) GSEA enrichment analysis of microglia subpopulations ( *p*＜0.05 ).


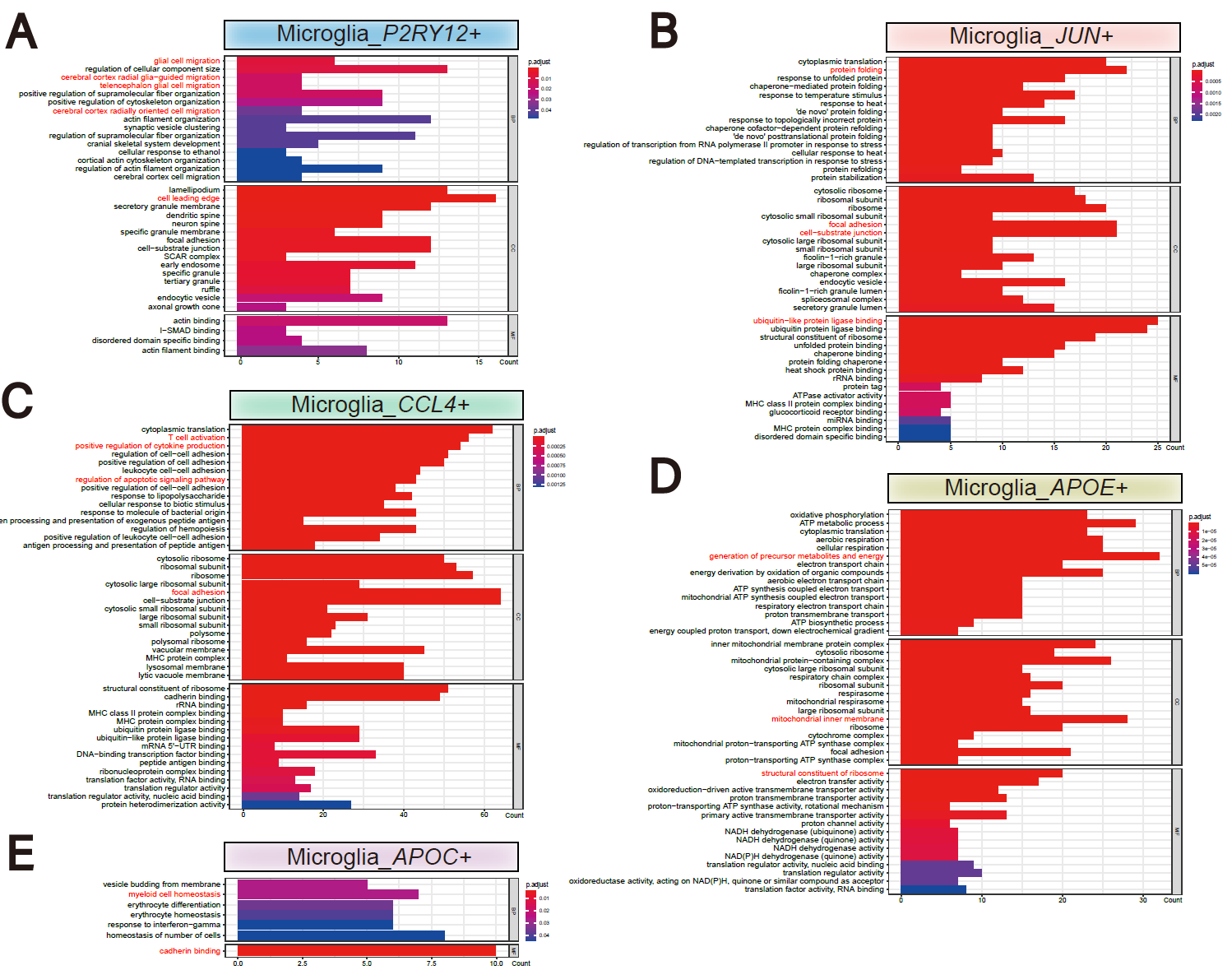


**Figure S4 GO enrichment analysis of different microglial subpopulations.**

(**A**) GO enrichment analysis of microglia_*P2RY12^+^* subcluster. (**B**) GO enrichment analysis of microglia_*JUN^+^* subcluster. (**C**) GO enrichment analysis of microglia_*CCL4^+^* subcluster. (**D**) GO enrichment analysis of microglia_*APOE^+^* subcluster. (**E**) GO enrichment analysis of microglia_*APOC^+^* subcluster.


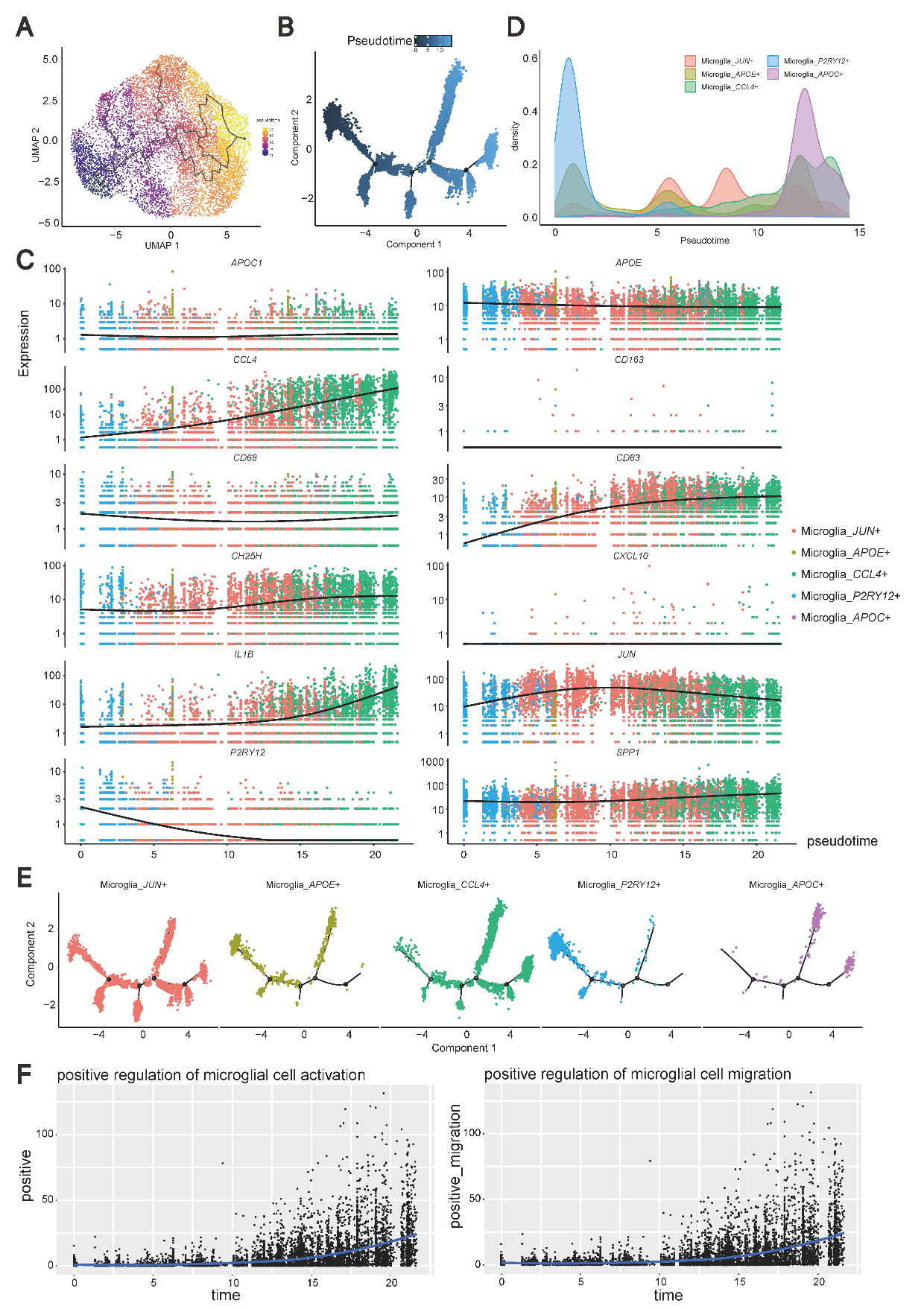


**Figure S5 Pseudotime analysis of different microglial subpopulations.**

(**A**) The pseudotime trajectory of the distribution in the UMAP graph. (**B**) The fate of cell developmental differentiation along a pseudotime trajectory. (**C**) The average expression changes over time for feature genes with regional expression characteristics on the pseudotime trajectory. (**D**) The change in the percentage of cells in each cell subtype over time on the pseudotime trajectory. (**E**) The distribution of five cell subpopulations in the cell differentiation fate trajectory. (**F**) The expression changes over time for gene sets with regional expression characteristics on the pseudotime trajectory: *CCL3*, *TAFA3*, *TTBK1*, *LRRK2*, *CTSC*, *etc.,* for ‘positive regulation of microglial cell activation’ and *CX3CR1*, *CCL3*, *P2RX4*, *CX3CL1*, *P2RY12*, *etc.,* for ‘positive regulation of microglial cell migration’.


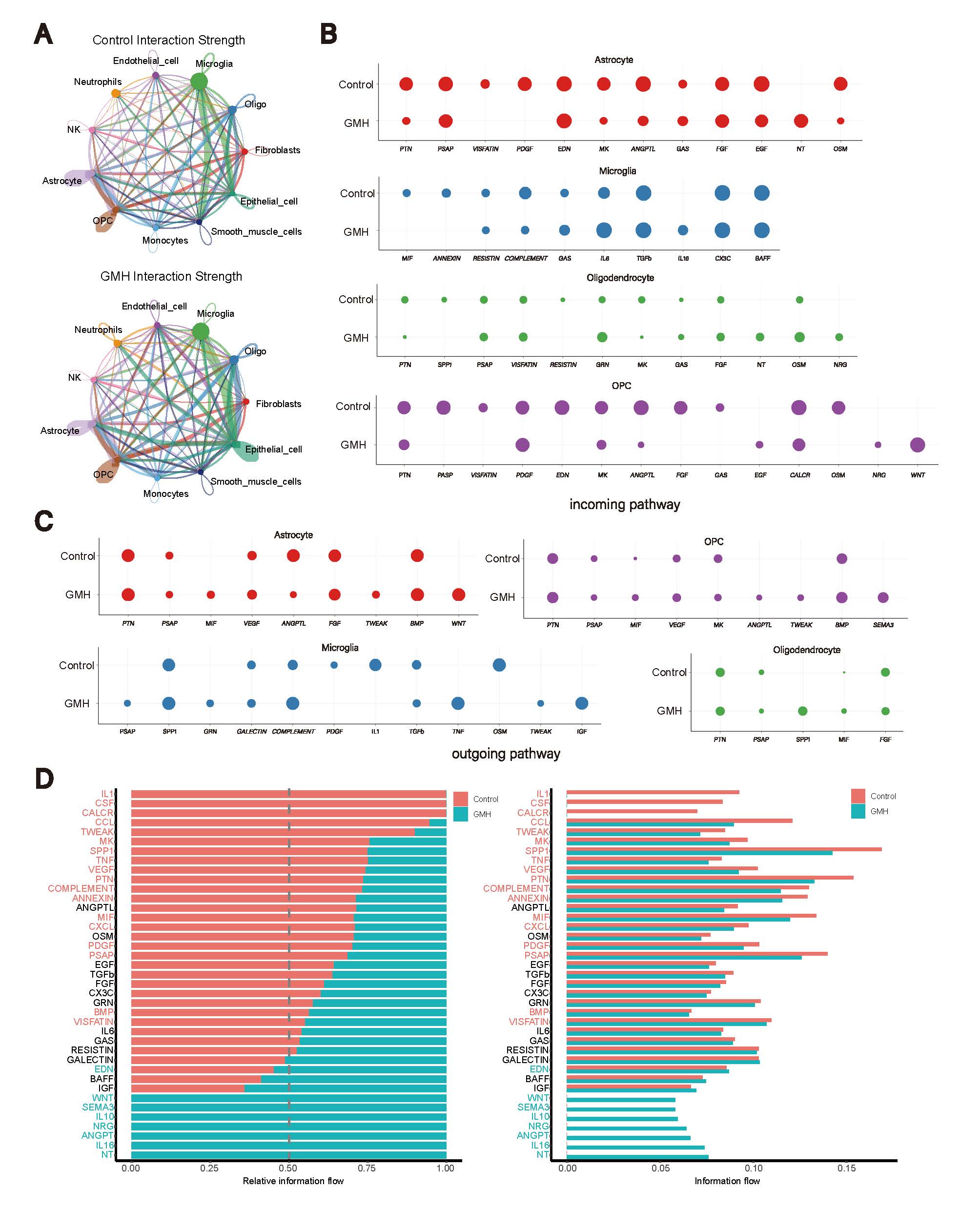


**Figure S6** **Differences in cellular communication between the experimental and control groups.**

(A) Demonstration of communication intensity between major cells in the experimental and control groups. (**B**) Differences in incoming cytokines of glial cells in the experimental and control groups. (**C**) Differences in the outgoing cytokines of glial cells in the experimental and control groups. (**D**) Comparison of cell-to-cell interactions between the GMH and control groups at the pathway level.

## Limitation

One of the limitations of our study is the sample scale. We acknowledge that a larger sample size would enhance the reliability of our findings. Despite our continuous efforts to increase the samples, it is essential to note that we applied strict inclusion criteria for patient selection, and patients with gray matter heterotopia requiring surgical resection in the same location are extremely rare. As a result, we proceeded with the analysis using the available two data points to provide timely results and contribute valuable insights to the field. We appreciate the opportunity to share our preliminary results in this journal through the Rapid Communication format, and we are fully committed to expanding our sample size to enrich the scientific literature further and advance the field of research.
